# Assessment of soil mulching field management, and deficit irrigation effect on productivity of watermelon varieties, and AquaCrop model validation

**DOI:** 10.1016/j.heliyon.2023.e21632

**Published:** 2023-10-26

**Authors:** Birara Gebeyhu, Genet Markos

**Affiliations:** Faculty of Water Resources and Irrigation Engineering, Arba Minch Water Technology Institute, Arba Minch University, Arba Minch, Ethiopia

**Keywords:** AquaCrop, Deficit irrigation, Productivity, Soil mulching, Watermelon varieties

## Abstract

Watermelon productivity in the Arba Minch irrigation scheme has been hampered by water scarcity, with only the Lady Bells watermelon variety being cultivated in the area. This challenge can be mitigated by adopting water-saving irrigation techniques and selecting water stress-resistant varieties. Hence, this study aimed to investigate the combined impact of soil mulching and deficit irrigation on the productivity of various watermelon varieties and validate the AquaCrop model. The experiment employed a randomized complete block design with two levels of water application (100 % SMD and 50 % SMD), two mulching practices (non-mulching and mulching), and four watermelon varieties: Lady Bells (V1), Green Pearl (V2), Kaolack (V3), and Koloss (V4). Soil physical properties and crop-related data were used to calibrate the AquaCrop model. Straw mulching, on average, conserved 64.50 mm and 262.75 mm of water under 100 % and 50 % water application levels, respectively. The minimum and maximum land productivity averages were 6.2 tonsha^−1^ (T13) and 17.6 tonsha^−1^ (T2), while water productivity ranged from 5.2 kgm^−3^ (T1) to 12.4 kgm^−3^ (T10). Lady Bell watermelon varieties displayed high sensitivity to water stress, with a 1.27 yield response factor under non-soil mulching treatment with 50 % water application. The mean benefit-cost ratio varied from 1.52 (T13) to 2.90 (T10). The average values of RMSE, NSE, and R^2^ for the AquaCrop model were 0.70, 0.65, and 0.80, respectively, indicating the model's acceptability in predicting the effects of mulching and deficit irrigation on watermelon productivity. Overall, the use of straw mulching combined with 50 % deficit irrigation, particularly for Green Pearl varieties, emerged as the most productive watermelon cultivation method in the Arba Minch region when facing limited irrigation water. Future research will focus on assessing the impact of deficit irrigation during various watermelon growth stages.

## 1 Introduction

Watermelon (*Citrullus lanatus*) is a very common fruit worldwide, especially in arid and semiarid climate environments [1]. Watermelons can survive desert climate regions when groundwater is available, and the mean optimum minimum and maximum base temperature for watermelon growth are about 18 °C and 35 °C respectively [2]. Watermelon (*Citrullus lanatus*) is a monoecious vine, a scrambling and trailing vine in the flowering plant family Cucurbitaceae [3]. The most adaptable watermelon varieties in Kenya, Nigeria, and Coastal Guyana were Yellow Crimson [4], Koloss [5], and Bonta [6], respectively. During the drought season, watermelons' fruit is used as a source of drinking water in portions of Sudan and Nigeria [7]. Deficit irrigation allows the farmer to stabilize the crop yield using less water when available irrigation water is insufficient to cover the full crop water requirement [8] and the most common method of irrigation application for watermelon crops is furrow [9]. Deficit irrigation has been widely investigated as a valuable, and sustainable production strategy in dry regions, and this practice aims to maximize water productivity [10]. Over the world, soil mulching in irrigation or rain-fed agriculture is used as water-saving technology in drought-prone areas [11] by creating favorable soil conditions [12]. In arid or semiarid regions, mulch has been used to conserve water through reduced evaporation while also increasing yields [13] and it is used to control weeds and reduce soil compaction [14]. The other role of soil mulching practice on agricultural land is to increase soil fertility [15] and that may have different thicknesses, colors, and raw materials [16]. Fruit, and vegetable production in water-limiting areas can be increased by adapting straw mulching field management with a 50–75 % deficit irrigation level [17]. The AquaCrop model was developed by the Food and Agricultural Organization (FAO) to predict the response of crop productivity to water stress, and it is designed to balance simplicity, robustness, and accuracy [18]. AquaCrop is a companion tool for a wide range of users, and applications including yield prediction under climate change scenarios [19]. AquaCrop includes sub-model components such as soil water balance; crop development, evaporative demand, and major agronomic practices such as irrigation and soil mulching [20]. The AquaCrop model is calibrated by using the length of the growing cycle, plant density, time to attain maximum canopy cover, time to begin canopy senescence, time to physiological maturity, time to start flowering, and duration of flowering [21].

Water scarcity is a major issue affecting watermelon production in Africa and the shortage of rainfall and depletion of soil moisture are key factors contributing to this problem [22]. In Arba Minch, Ethiopia, water scarcity has led to conflicts among water users. The availability of rainfall in the region is inconsistent and often insufficient for agricultural needs. This variability in rainfall patterns further complicates watermelon production. Due to water scarcity and inadequate soil moisture, the productivity of watermelon in the Arba Minch irrigation scheme was low. This means that farmers are not able to achieve high yields from their crops. In the area, only one watermelon variety, the lady bells variety, is being practiced. However, this variety seems to have issues with its leaf coloration (yellowish), and a significant portion of its production is non-marketable. Predicting watermelon productivity under conditions of water scarcity and soil mulching field management is challenging, especially on large agricultural land. This suggests a need for better techniques and tools to manage water resources effectively and optimize crop yields.

To minimize the impact of water scarcity, the use of water-saving irrigation technologies like mulching and deficit irrigation is proposed [23]. These techniques can help optimize water usage in agriculture, especially in regions with limited water resources. It is mentioned that selecting water-stress-resistant watermelon varieties can improve the productivity of watermelon fruit under limited water supply [24]. This suggests that choosing the right crop varieties that can thrive in water-scarce conditions is essential. The AquaCrop model is mentioned as a tool to accurately predict the response of watermelon productivity to soil mulching and deficit irrigation. This model can be valuable in planning and managing watermelon cultivation to maximize yields with minimal water usage. Up to the time of the study, there had been no research conducted in the Arba Minch area to introduce water-saving irrigation technology, water-stress-tolerant watermelon varieties, or to validate the AquaCrop model. This indicates a need for research and interventions to address water scarcity challenges in watermelon farming. Therefore, this study was conducted to assessing the combined impacts of soil mulching and deficit irrigation on watermelon productivity, identifying water-stress-tolerant watermelon varieties, and validating the AquaCrop model. These efforts aim to find ways to increase watermelon yield while conserving water resources and promote watermelon agriculture through the use of straw mulching material.

## 2 Material and methods

### 2.1 Location of experimental site

The experimental site was situated in the southern region of Ethiopia, approximately 455 km from the capital city, Addis Ababa. It was positioned at coordinates 6° 4′2.44″N latitude and 37°34′0.77″E longitude, with an average elevation of 1200 m above sea level. More specifically, the experiment was conducted within the demonstration farmland of Arba Minch University, located in the Arba Minch Zuria woreda district. The primary crops cultivated in this experimental area included tomatoes, watermelons, peppers, onions, maize, permanent bananas, and mangoes.

### 2.2 Climate

**Fig. 1:**
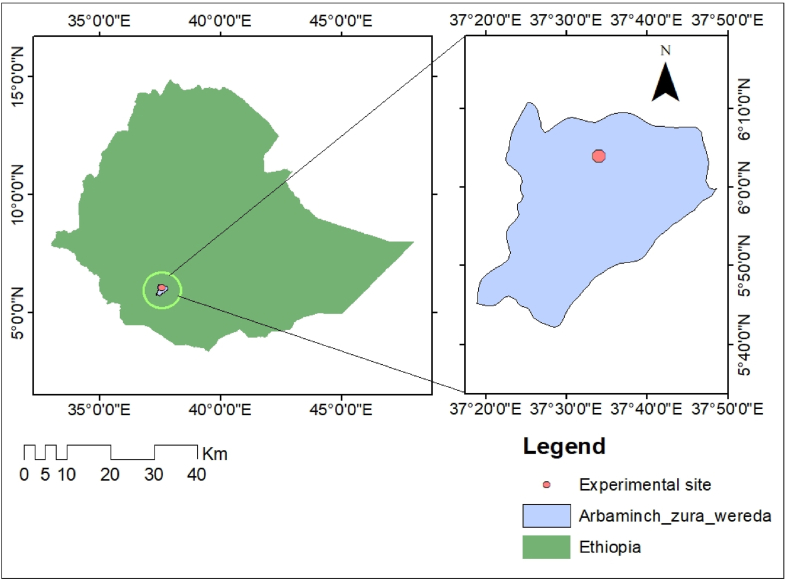
Location of experimental site.

**Fig. 2:**
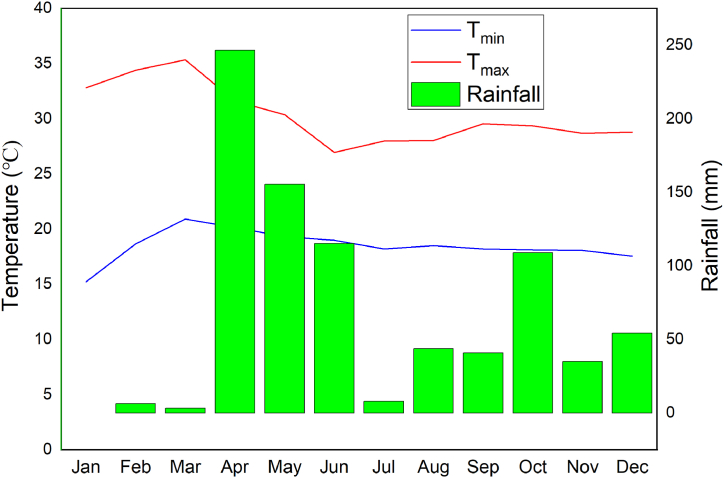
Average monthly minimum temperature (T_min_), maximum temperature (T_max_), and rainfall.

According to the climate description, the region's driest months are January through March and July through September are relatively dry (see Fig. 1). According to the agro-ecological climate zone classification, the research area fell within the kola climate zone. The study area exhibited an average minimum temperature of 18 °C and a maximum temperature of 29.3 °C. Additionally, it received an average annual rainfall of 890 mm. The rainy season in this region typically spanned from April to June, with the lowest rainfall occurring in January (Fig. 2). Notably, the area experienced its highest monthly rainfall in April, gradually tapering off from July onward. As per the climate description, the driest months in the region were January through March, while July through September were relatively dry periods.

### 2.3 Treatment replication & experimental design

The treatments were laid out in Randomized Complete Block Design (RCBD) with two water level applications (100SMD, and 50%SMD), two filed management techniques (Mulching and non-mulching), and four watermelon varieties such as lady bell (control variety), green pearl, Kaolack, and Koloss varieties. Experiments were sixteen treatments with three replications as coding (Table 1).

**Table 1:** Treatment of experimental plot.

TR	varieties	FM	Applied % SMD	TR	Varieties	FM	Applied %SMD
T1	V1	M	100	T9	V1	M	50
T2	V2	M	100	T10	V2	M	50
T3	V3	M	100	T11	V3	M	50
T4	V4	M	100	T12	V4	M	50
T5	V1	NM	100	T13	V1	NM	50
T6	V2	NM	100	T14	V2	NM	50
T7	V3	NM	100	T15	V3	NM	50
T8	V4	NM	100	T16	V4	NM	50

V1, V2, V3, and V4 were lady bells, green pearl, Kaolack, and Koloss watermelon varieties respectively, and, TR = Treatments, M = mulching and NM = none mulching, and FM = field management.

**Fig. 3:**
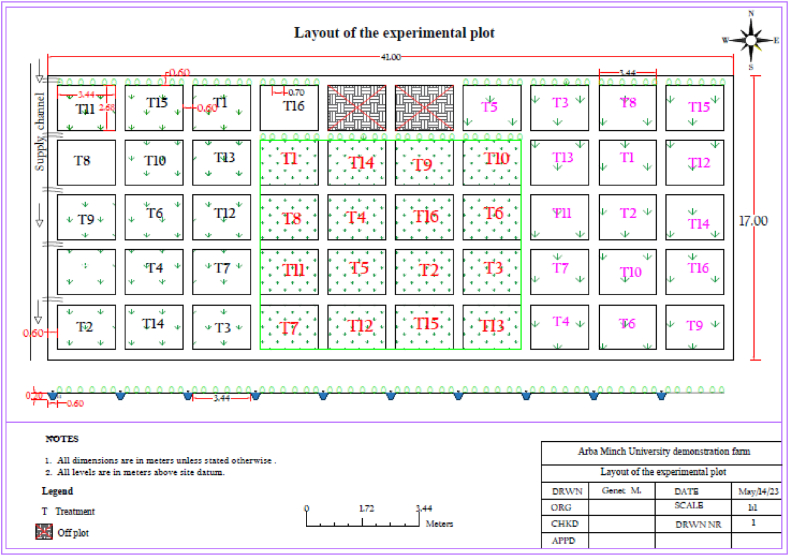
Alignment of the experimental plot.

For all the furrow approaches in the study, the field plot size was 3.44 by 2.68 m, with a 0.6-m free space between blocks and plots (as shown in Fig. 3). The crop spacing for the experiment was set at 0.7 m, and the furrows had a trapezoidal cross-section. Specifically, the experimental furrow had a waterway depth of 60 cm and a furrow depth of 10 cm. In the treatment, Teff straw was utilized as the mulching material, but it covered only 25 % of the total plot area. This means that the mulching was concentrated primarily within the fruit root zone.

### 2.4 Soil physicochemical properties

An undisturbed soil sample was collected at 80 cm soil depth with a 20 cm depth interval by using Auger, and the height, and diameter of the sampler core were 6 cm, and 5.5 cm, respectively, and it was dry for 24 h at 105°c. Soil field capacity and permanent wilting point were evaluated through a pressure plate apparatus and soil texture also evaluated through a hydrometer test at Arba Minch University soil mechanics laboratory. The bulk density of the soil was expressed [25,26] and it was used to determine the volumetric water content of the soil after estimated by Eq (1).

(1) $$\text{Bulk}\;\text{density}\;\left(\rho b\right)=\frac{\text{Mass}\;\text{of}\;\text{dry}\;\text{soil}}{\text{Total}\;\text{volume}\;\text{of}\;\text{soil}\;\text{sample}}$$

Based on [27], soil electrical conductivity (ECe) and pH value were determined using potentiometric and conductometric methods, respectively, with a soil-to-water volume ratio of 1:2.5. Organic matter was also measured in Arba Minch University's chemistry laboratory using edita titration. At the start of the experiment, the soil infiltration rate was determined using a double-ring infiltrometer, which was used to manage irrigation application rate.

### 2.5 Irrigation scheduling

**Fig. 4:**
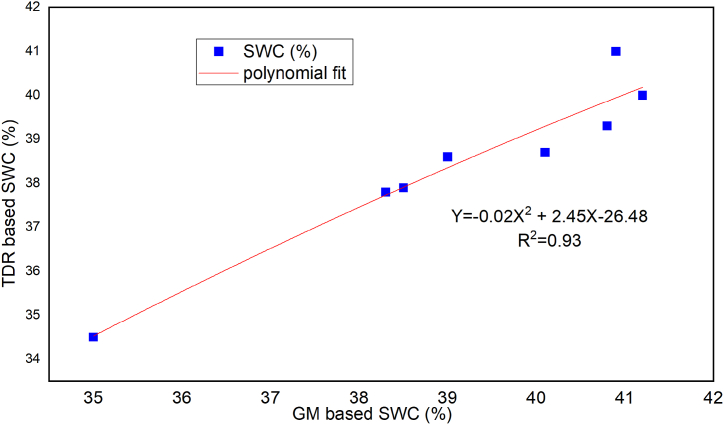
Calibrated equation of TDR with GM

Soil moisture depletion (SMD) is the difference between field capacity and the actual moisture in the soil root zone at any given time before irrigation and it is the amount of water required to bring the soil in the root zone to field capacity. A time-domain reflectometer (TDR) is an electrical device that assesses soil water content indirectly by propagating electromagnetic waves into the soil profile [28]. The gravimetric direct soil water measurement involves taking a soil sample, weighing, oven drying, and reweighing it, and then expressing the moisture content as a percentage of the oven-dry weight of soil [29]. Then gravimetric soil water content was evaluated by dividing the mass of water by the mass of dry soil. As shown in Fig. 4, the soil water content was measured using a time dominates reflectrometer (TDR) that had been calibrated using the gravimetric method (GM).

Where; TDR = time dominates reflectrometer, SWC = soil water content, GM = gravimetric method, vol = Volume-based.

The effective root depth of the crop corresponding with the growth stage was measured directly at the time of irrigation. Irrigation was applied when the ratio of the depth of soil water depletion to the depth of soil water at field capacity was equal to management allowable depletion (MAD), and the depth of soil water depletion was calculated through Eq (2).

(2) $$D\text{epth}\;\text{of}\;\text{soil}\;\text{water}\;\text{depletion}\;\left(\text{mm}\right)=\sum_{i=1}^{n}\left(\frac{\Theta c-\Theta i}{100}\right)i*\text{Di}$$

SMD = soil moisture depletion in the root zone prior to irrigation (mm), ө_c_ = field capacity of the soil, (%), ө_i_ = soil water content before the irrigation (%), Di = ith layer of crop root depth (mm) and n = number of layers in the root zone.

Applied discharge to the furrow at the time of irrigation was measured by using an RBC flume. Leaf length (L), and width (W) were also measured manually with a tape meter that is used to calculate field-based leaf area, leaf area index, and canopy cover. Based on [30], total leaf area A (cm^2^), and leaf area index were estimated through Eq (3) &4, respectively. Field-based canopy cover was also evaluated based on [31] as described in Eq (5).

(3) $$\text{Total}\;\text{leaf}\;\text{area}=0.759*\sum_{n=1}^{n}\left(\text{WL}\right)$$

(4) $$\text{Leaf}\;\text{area}\;\text{index}=\frac{Measured\;area\;per\;plant*number\;of\;plant}{Ground\;area}$$

(5) $$\text{Canopy}\;\text{cover}=1.005*{\left[1-\exp\left(-0.61\text{LAI}\right)\right]}^{1.2}$$

### 2.6 Yield response factor

The yield response factor (K_y_) indicates the reduction in crop yield as a function of evapotranspiration and can be an important tool for yield forecasting and it was estimated based on [32] by using Eq (6).

(6) $$\frac{Ym-Ya}{Ym}=Ky\frac{\left(ETm-ETa\right)}{ETm}$$

where; *Y*_*m,*_ Y_a_, ET_m_, and ET_a,_ were maximum crop yields (tonha^−1^), actual crop yield (tonha^−1^), maximum evapotranspiration (mm), and actual evapotranspiration (mm), respectively.

### 2.7 AquaCrop model description

The AquaCrop model is a water-driven model that simulates the crop yield response to water [33], and climate data such as temperature, relative humidity, rainfall, and crop data such as days of emergence were used as input. According to Ref. [34], the AquaCrop model predicted watermelon biomass, fresh yield, and canopy cover through Eq (7), Eq (8), and Eq (9) or Eq (10), respectively.

(7) $$\text{Biomass}\left(\frac{ton}{ha}\right)=Wp\sum Tr$$

(8) $$\text{Fresh}\;\text{yield}\;\left(\frac{ton}{ha}\right)=B*HI$$

(9) $$CCt=CCo*e^{tCGC}$$

,ifCC≤CCx/2

(10) $$CCt=CCx-0.25\frac{{\left(CCx\right)}^{2}}{CCo}e^{-tCGC}$$

,ifCC>CCx/2

where; CC_t_ = canopy cover at time t (fraction ground cover), CC_o_ = initial canopy size at t = 0 (fraction ground cover), CC_x_ = maximum canopy cover (fraction ground cover), CGC = canopy growth coefficient (an increase of fraction ground cover per day) and t time (day).

Soil salinity and surface runoff were not incorporated in model calibration, although optimal weeding management conditions and the optimum level of soil fertility (90 %) were considered in all treatment simulations. The AquaCrop model was calibrated for the period from February 4, 2022, to May 24, 2022 (season 1) and October 23/2022 to February 3/2023 (season 2) under crop calibration parameter (Table 2).

(11) $$\text{RMSE}=\sqrt{\frac{1}{n}\sum_{i=1}^{n}(Si-\text{Mi})ˆ2}$$

(12) $$\text{NSE}=1-\frac{\sum_{i=1}^{n}(Si-\text{Mi})ˆ2}{{\sum_{i=1}^{n}\left(\text{Mi}-\text{Mav}\right)}^{2}}$$

where S_i_*,* M_i_*,* and M_av_ were simulated value, measured values and mean of measured values, respectively, and n is the number of observations.

**Table 2:** AquaCrop calibration parameter.

Description	Value	Unit
Base temperature	10	ͦc
Upper temperature	35	ͦc
Growing cycle	110	Days
Initial canopy cover	1.5	%
Mode of planting	Sowing	–
Maximum canopy cover	90	%
Plant density	2500	Plantsha^−1^
Day one to full emergency	7	Days
Day one to maximum canopy	50	Days
Day one senescence	80	Days
Day one harvesting	110	Days
Maximum effective depth	0.8	m
Root growth rate	1.5	cmday^−1^
Normalized water productivity	31	gm^−3^
Reference harvesting index (HIo)	50	%
Crop genetic	C_3_	–
Indicative range for climate and CO_2_ normalized (wp*)	30–35	gmcm^−2^

The standard deviation of the residuals (prediction errors), known as the root means square error (RMSE), is zero when the simulation value and the measured value agree, indicating strong model performance [35]. Nash–Sutcliffe coefficient of efficiency (NSE) was used to assess the predictive performance of the AquaCrop model and based on [36], RMSE and NSE were calculated by using Eq (11), and Eq (12).

## 3 Result and discussion

### 3.1 Soil physicochemical properties

The soil textural class of the experimental site was clay, and the average bulk density of soil (BD) was also 1.26gmcm^−3^. The soil was suitable for agriculture practices to be uncompacted with a bulk density less or equal to 1.63gmcm^−3^ [37]. Therefore, the bulk density of the current study was found with the recommended value which means the experimental site was too suitable for agriculture practice with normal soil compactness status. The average soil organic matter (OM), extracted electric conductivity (EC_e_), and soil pH values were 0.84 %, 0.13dsm^−1^, and 7.74, respectively. According to Ref. [38], the estimated value for electric conductivity demonstrates that the soil has no issues with salinity and is ideal for growing fruits. Mean soil field capacity (FC), and permanent wilting point (PWP) were 38.95 %, and 26.68 %, respectively (Table 3). The initial and basic infiltration rates of the experimental area were 1.40mmmin^-1^, and 0.08mmmin^-1^, respectively. In order to reduce runoff issues on fields, the application rate of soil was governed by the predicted basic infiltration rate of the soil, which meant that the rate of irrigation was not more than the basic infiltration rate. The average value of irrigation application rate during the experiment was 0.075mmmin^-1^, which was less than basic soil infiltration rate.

**Table 3:** Soil physiochemical properties.

Depth (cm)	% clay	% Silt	% sand	Texture	BD (gmcm^−3^)	OM (%)	EC_e_ (dsm^−1^)	pH (−)	FC (%vol)	PWP (%vol)
0–20	43.00	31.00	26.00	Clay	1.23	0.87	0.12	7.75	39.30	26.00
20–40	44.00	23.40	32.60	Clay	1.24	0.86	0.13	7.78	39.30	26.70
40–60	44.50	22.20	33.30	Clay	1.30	0.82	0.11	7.76	37.70	27.30
60–80	44.40	25.00	30.60	Clay	1.28	0.79	0.14	7.80	39.50	26.70
Average	44.00	25.40	30.60	Clay	1.26	0.84	0.13	7.77	38.95	26.68

### 3.2 Mulching effect soil water availability

**Fig. 5:**
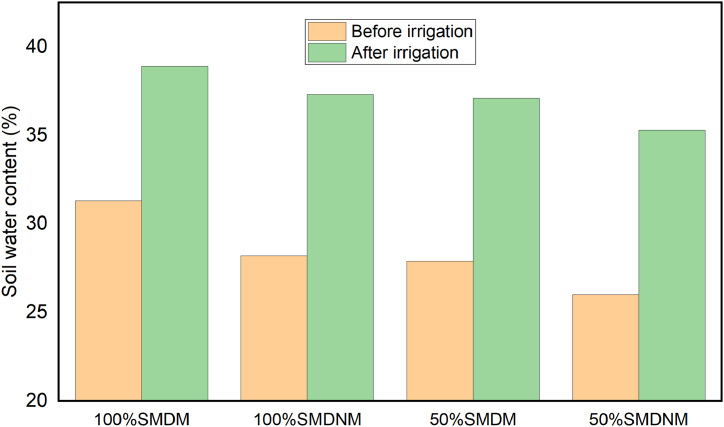
Effects of treatment on average soil water content before, and after irrigation.

In comparison to non-mulching practices, straw mulching with full water application increased soil water storage in the crop root zone by 10 % [39]. Straw mulching was used as a field management approach in the current study, and it increased soil water storage by 5.10 % and 10.9 % for full and half water applications, respectively. In general, the average soil water content under the mulching treatment was highest before and after irrigation as compared to the non-mulching treatment (Fig. 5).

Where; SMD=Soil moisture depletion, M = mulching and NM = none mulching.

Under full, and half-level water application, soil mulching treatment saved 64.50 mm, and 262.75 mm of water, respectively, and this amount of irrigation water may be used to irrigate an extra area. This anticipated water savings for irrigation was based on the highest quantity of water typically consumed in full water level applications without mulching practices treatment (461 mm) (Table 4). Total water consumption in season 2 was maximum compared with season 1 due to the availability of effective rainfall in season 1. Total water consumption during season 1 (February 4/2022 to May 24/2022), and in season 2 (October 23/2022 to February 3/2023) was summarized in Table 4. Soil mulching field management practices could save irrigation water requirements approximately 40 % [40]. Soil mulching field management practice under the half level of water application in the current study saved 57 % of irrigation water compared with full level of water application without mulching.

**Table 4:** Total seasonal actual evapotranspiration (ETa) under different treatments (TR) and saving water.

	Season 1	Season 2				Season 1	Season 2		
TR	ETa (mm)	ETa (mm)	Average (mm)	Water saving (mm)	TR	ETa (mm)	ETa (mm)	Average (mm)	Water saving (mm)
T1	386.50	406.50	396.50	64.50	T9	193.25	203.30	198.25	262.75
T2	386.50	406.50	396.50	64.50	T10	193.25	203.30	198.25	262.75
T3	386.50	406.50	396.50	64.50	T11	193.25	203.30	198.25	262.75
T4	386.50	406.50	396.50	64.50	T12	193.25	203.30	198.25	262.75
T5	450.00	472.00	461.00	0.00	T13	225.00	236.00	230.50	230.50
T6	450.00	472.00	461.00	0.00	T14	225.00	236.00	230.50	230.50
T7	450.00	472.00	461.00	0.00	T15	225.00	236.00	230.50	230.50
T8	450.00	472.00	461.00	0.00	T16	225.00	236.00	230.50	230.50

### 3.3 Irrigation depth and scheduling

**Fig. 6:**
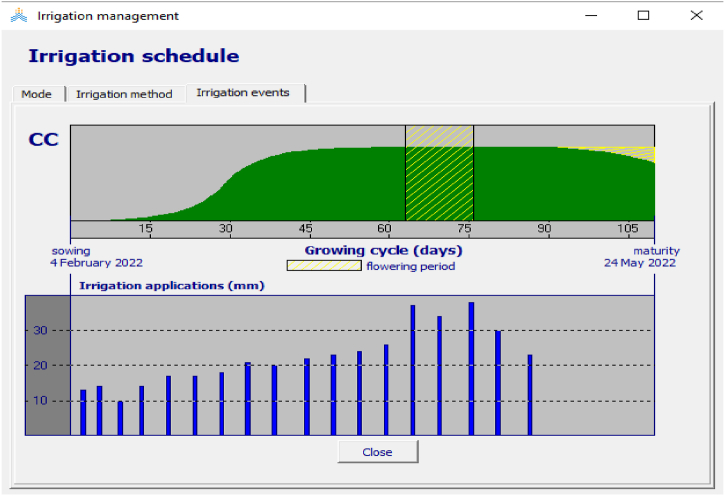
AquaCrop based irrigation scheduling under mulching and full irrigation application treatment.

The depth of irrigation requirement varied with the growth stage of crop and field management practice. The total number of irrigations during the growing period was 18 and 22, in season 1 (Fig. 6**),** and season 2, respectively. The average number of irrigation intervals was 6 days, and the interval of irrigation was similar with each treatment only amount irrigation varied with deficit level and mulching practices.

### 3.4 Watermelon agronomic performance

#### 3.4.1 Steam length, branch and canopy cover

**Fig. 7:**
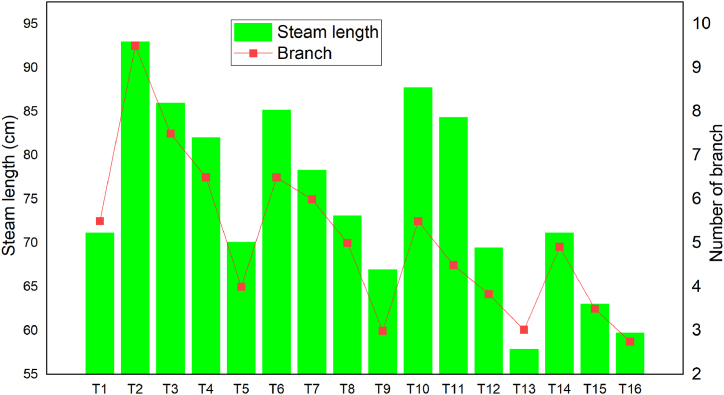
Average steam length, and number of branches.

The average steam length and number of branches of watermelon cultivars decreased by 23 % in the second phase of treatment due to uncontrolled watermelon diseases such as Fusarium wilting. This disease was an underground soil-borne disease that affects watermelon roots. The mean maximum steam length and number of branches were observed under treatment 2, and the minimum was also observed under treatment 13 (Fig. 7). Based on [41], teff straw mulching with full level of water application could increase the number of branches per crop by 49 %, and in the current studies straw mulching was increased by 45.50 % compared with control treatment (T5).

**Fig. 8:**
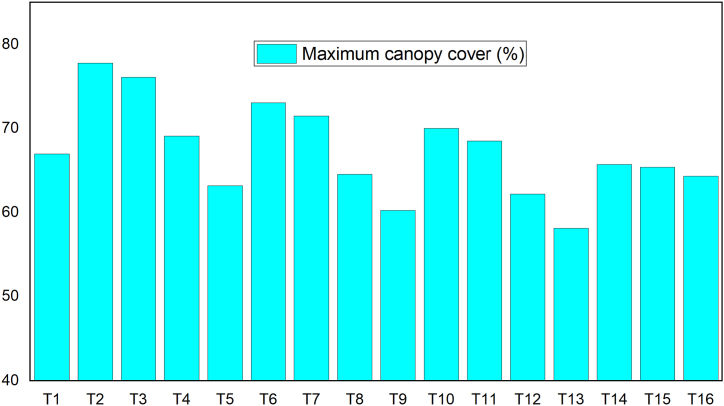
Average maximum canopy cover for each treatment.

According to Ref. [42], the maximum canopy cover of watermelon under optimum soil moisture, good field management, and disease protection ranges from 80 to 90 %. The average maximum canopy cover of watermelon varieties under each treatment was varied from 58.10 (T13) to 77.80 % (T2) it was not found within recommended values due to crop disease, especially in season 2 (Fig. 8). In the case of all treatments, the maximum and minimum value canopy cover were observed under green pearl, and lady bell (control) varieties. This result showed that even with proper watering and mulching, the lady bell watermelon cultivar (control) had less canopy cover than other kinds in the area.

#### 3.4.2 Fruit length and diameter

The mean fruit length displayed a range between 25.60 cm (T13) and 45.0 cm (T2), while fruit diameter exhibited variation from 15.9 cm (T13) to 25.2 cm (T2) (as indicated in Table 5). Across all treatments, the lowest and highest values for fruit length and diameter were recorded for green pearl watermelons (V2) and lady bell watermelons (V1), respectively. Particularly, when subject to full-level water application, green pearl watermelons (V2) yielded an average fruit length of 27.38 cm and a fruit diameter of 38.14 cm, as reported in a prior study [43]. These measurements significantly diverged from the findings of the current investigation. In the present study, the implementation of soil mulching field management practices combined with a full level of water application resulted in a substantial enhancement of watermelon fruit length and diameter, increasing them by 15 % and 15.10 %, respectively.

**Table 5:** Effects of treatment on watermelon fruit length, and diameter.

Treatment	Fruit length (cm)	Fruit diameter (cm)	Treatment	Fruit length (cm)	Fruit diameter (cm)
T1	34.30	21.30	T9	28.50	17.70
T2	45.00	25.20	T10	37.40	20.90
T3	44.20	24.00	T11	36.70	19.90
T4	38.00	22.60	T12	31.50	18.80
T5	29.80	18.50	T13	25.60	15.90
T6	39.20	21.90	T14	33.60	18.80
T7	38.50	20.90	T15	33.00	17.90
T8	33.10	19.70	T16	28.40	16.90

**Fig. 9:**
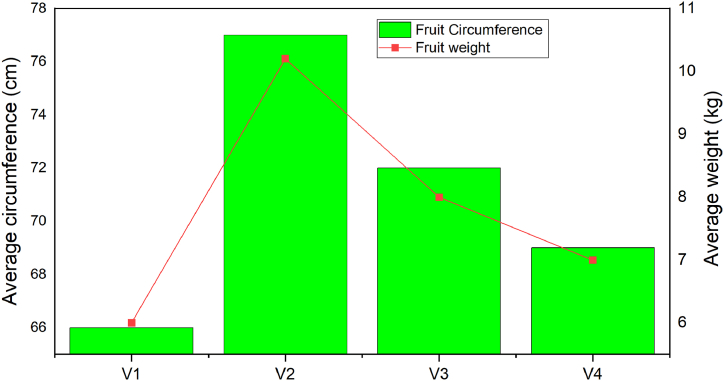
Average watermelon fruit circumference and weight.

The average weight of watermelon fruit under optimum soil water content and field management was 7.2 kg [44] which was less than compared with the current study. The average weight of the current study was 8 kg under full water level application (Fig. 9). The average weight of lady bell watermelon varieties ranges from 5.8 to 8.8 kg [45], and the average value of the current study was 6 kg. Within certain water deficit limits, irrigation practices do not greatly affect the number of fruits per plant but affect the fruit size, shape, weight, and quality [46]. All above agronomic performance of watermelon varieties such as fruit diameter, length, circumference and weight in season 2 averagely decreased by 30 % compared with season 1. The typical minimum and maximum fruit weight and circumference were noted for the lady bell and pearl watermelon types, respectively, and small-hold farmers in the region can be recommended to plant green pearl watermelon cultivars to increase its product.

#### 3.4.3 Land and water productivity

**Fig. 10:**
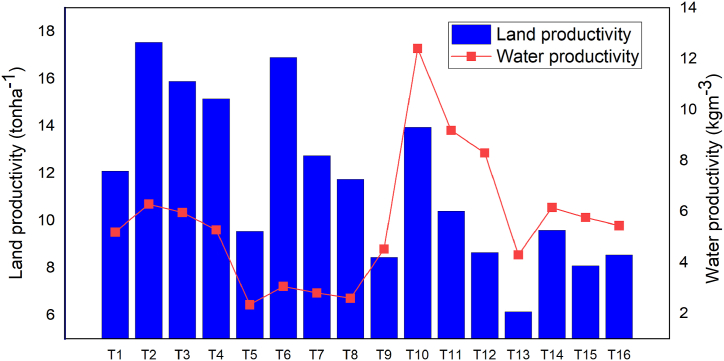
Average land and water productivity.

According to Ref. [47], the range of water productivity for watermelon (*Citrullus lanatus*) under non-water stress conditions was between 2.7 and 14.33 kgm^−3^. Under full-level water application, the water productivity of watermelon varies from 5 to 8 kgm^−3^ [46]. In the Arba Minch climatic zone, full-level water application without soil mulching field management techniques resulted in an average water productivity of watermelon types of 5.7 kgm^−3^, which was found within the previously mentioned range. Straw mulch significantly increased crop production [48], and the use of straw mulch as an in situ water harvesting technique would further help in the long term to mitigate nutrient losses from the soil due to the release of nutrients from the straw. The potential yield of watermelon fruit ranges from 7.39 to 58.49 tonsha^−1^ under sufficient rainfall and optimum soil fertility [49]. The average land productivity of watermelon under full-level water application also varied from 10 to 35 tonsha^−1^ [46]. The average maximum land productivity of green pearl watermelon varieties in Ethiopia under full water level application with straw mulching was 24.59 tonsha^−1^ [50]. The average maximum land productivity of the current study under similar treatment was 17.6tonsha^−1^ (Fig. 10). Soil mulching highly increased the land productivity of watermelon under full and half water level application compared with non-mulching treatment. This significant change in yield production was observed under 25 % soil mulching field management practices that show the surface of mulching increases yield production also increase more and more. Under full-level water application, soil mulching field management strategy increased watermelon output by 19.10 %, and under half-level water application, by 27.90 %. This reveals that soil mulching field management techniques have a very favorable effect when there is insufficient irrigation.

### 3.5 Yield response factor

The maximum production (Ym) and water used (ETm) were 17.6 tonsha^−1^, and 461 mm, respectively. The total water requirement of watermelon under the full level of water application without mulching treatment varies from 365.20 to 480.00 mm [51]. According to Ref. [46], the water requirement of watermelon varieties from 400 to 600 mm without any water conservation practices, and based on [52], the average water requirement of watermelon was 343 mm. The total water requirement of watermelon in the Arba Minch climate zone was also found within the recommended range and the water stress yield response factor for full level of water application with soil mulching field management practices was not considered. Based on [44], Ky greater than 1 implies that the crop is very sensitive to water deficits, Ky less than 1 means that it is more tolerant to water deficits, and Ky equal to 1 corresponds to a direct proportion of yield reduction to reduced water use. Yield response factors were estimated for half water level application under mulching and non-mulching field management practices. Lady bell watermelon varieties were relatively sensitive to water stress with a 1.27 yield response factor under non-soil mulching treatment with the half water application level (Table 6). The average watermelon yield response factor under 50 % of water deficit without soil mulching field management practices was 1.26 [44] it was an approach to the value of the current study. Therefore, lady bell (V1), and Kaolack (V3) watermelon varieties were highly sensitive to water deficit. Generally, the most adaptable watermelon varieties under all treatments were green pearl watermelons with less value of yield reduction response factor for the Arba Minch climate zone.

**Table 6:** Average watermelon varieties yield response factor (K_y_).

Treatments	Yield (ton ha^−1^)	ET_a_ (mm)	1-(Y_a_/Y_m_)	1-(ET_a_/ET_m_)	K_y_
100%SMDV1NM	9.6	461	0.43	0.00	0.00
100%SMDV2NM	16.9	461	0.00	0.00	0.00
100%SMDV3NM	12.8	461	0.25	0.00	0.00
100%SMDV4NM	11.8	461	0.30	0.00	0.00
50%SMDV1M	8.5	198.25	0.50	0.57	0.88
50%SMDV2M	14.0	198.25	0.17	0.57	0.31
50%SMDV3M	10.4	198.25	0.38	0.57	0.67
50%SMDV4M	8.7	198.25	0.49	0.57	0.86
50%SMDV1NM	6.2	230.5	0.64	0.50	1.27
50%SMDV2NM	9.6	230.5	0.43	0.50	0.86
50%SMDV3NM	8.1	230.5	0.52	0.50	1.04
50%SMDV4NM	8.6	230.5	0.49	0.50	0.99

ETa, Y_a_ 1-(Y_a_/Y_m_), and 1-(ET_a_/ET_m_) were actual crop evapotranspiration (mm), actual harvesting yield (tonha^−1^), relative yield decrease, and relative evapotranspiration deficit, respectively.

### 3.6 Benefit-cost ratio

**Fig. 11:**
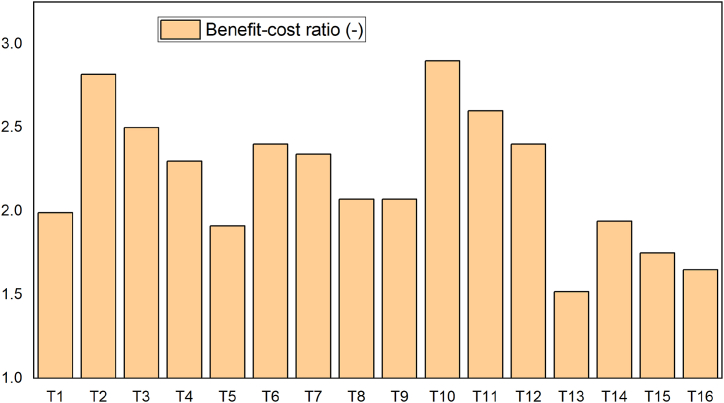
Average benefit-cost ratio analysis.

The average benefit-cost ratio of watermelon fruit under full irrigation with soil mulching was 2.50 [50], which was greater than the value of the current study, which was 2.40. This shows that the productivity of watermelons varied with soil type, climate, and disease effects. The minimum and maximum values of the benefit-cost ratio of the experiments were 1.52 (T13) and 2.90 (T10), respectively (Fig. 11). This study shows that the net benefit of watermelon agriculture in the arba minch area could be maximized in 50 % deficit irrigation with straw mulching water conservation practices, with green pearl (V2) being the most productive watermelon for this treatment. As a result, the arba minch irrigation scheme and the smallholders nearby have adapted this variety rather than the control variety (lady bell).

### 3.7 Model performance

**Fig. 12:**
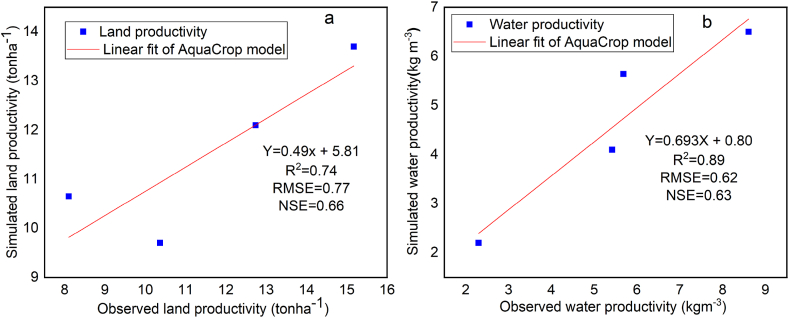
AquaCrop model validation for land productivity (a) and water productivity (b).

Four treatments were used for model validation, such as full and half water level application with mulch, and without mulch treatment. RMSE measures the magnitude of the difference between simulated and observed values and ranges from 0 to positive infinity, with 0 indicating good and infinity indicating poor model performance [53]. The average value of the root mean square error (RMSE) of the current study was 0.7, found between 0 and 7, which was within the acceptable range suggested by Ref. [54]. The mean coefficients of determination, or correlation coefficients (R^2^), of the current studies were 0.82, which was found within the recommended range value of 0.60 and 1.00. The Nash–Sutcliffe coefficient of efficiency (NSE) varies from -∞ to 1 where 1 indicates a perfect match between the model estimates and the observations. NSE in the range of 0.66–0.74, indicating acceptable model performance in simulation [53], and the value of the current study was found with the recommended value as presented in Fig. 12. Therefore, the AquaCrop model was suitable to simulate both land and water productivity with soil mulching and deficit irrigation treatment.

## 4 Conclusion and recommendation

The study aimed to investigate the impact of deficit irrigation and straw mulching on various watermelon varieties while validating the AquaCrop model. Applying a 50 % deficit irrigation level with straw mulching was found to be an effective method for conserving water during the growing season. This approach has the potential to increase the amount of cultivable land per season while managing limited water resources efficiently. Among the watermelon varieties studied, the green pearl variety consistently exhibited the highest productivity, while the lady bell variety had lower productivity. This information can guide farmers in choosing the most suitable watermelon variety for their specific conditions. Lady bell watermelon varieties showed the most significant reduction in yield when subjected to a 50 % deficit irrigation level without straw mulching. This underscores the importance of tailoring irrigation strategies to the specific needs of different watermelon varieties. The combination of half-level water application with straw mulching and green pearl watermelon varieties emerged as the most water-saving and productive irrigation practice. It also yielded the highest benefit-cost ratio, making it an economically viable choice for small hold farmers in the Arba Minch districts. The study successfully validated the AquaCrop model's accuracy in predicting watermelon productivity in semi-arid regions with water deficits and straw mulching practices and it provides confidence in the model's utility for optimizing irrigation strategies. Due to cost limits and treatment complexity, the sensitivity of watermelon productivity to water deficit differed with early, development, mid, and late growing phases, and it was not investigated. As a result, future researchers will assess the impacts of deficit irrigation at each stage of growth, which is crucial for improving watermelon production while decreasing water consumption. In summary, this study offers valuable insights into sustainable water management and watermelon cultivation practices in semi-arid regions. It emphasizes the importance of varietal selection, efficient irrigation methods, and the use of predictive models like AquaCrop to enhance agricultural productivity while addressing water scarcity challenges.

## Data availability statement

Data will be made available on request.

## Additional information

No additional information is available for this paper.

## Declaration of competing interest

The authors declare that they have no known competing financial interests or personal relationships that could have appeared to influence the work reported in this paper.
